# Development of Antioxidant and Nutritious Lentil (*Lens culinaris*) Flour Using Controlled Optimized Germination as a Bioprocess

**DOI:** 10.3390/foods10122924

**Published:** 2021-11-25

**Authors:** Daniel Rico, Elena Peñas, María del Carmen García, Dilip K. Rai, Cristina Martínez-Villaluenga, Juana Frias, Ana B. Martín-Diana

**Affiliations:** 1 Subdirection of Research and Technology, Agro-Technological Institute of Castilla y León, Consejería de Agricultura y Ganadería, Finca de Zamadueñas, 47171 Valladolid, Spain; ricbarda@itacyl.es (D.R.); ita-gargutma@itacyl.es (M.d.C.G.); 2 Department of Food Characterization, Quality and Safety, Institute of Food Science, Technology and Nutrition (ICTAN-CSIC), 28006 Madrid, Spain; elenape@ictan.csic.es (E.P.); c.m.villaluenga@csic.es (C.M.-V.); frias@ictan.csic.es (J.F.); 3 Department of Food BioSciences, Teagasc Food Research Centre Ashtown, 15 Dublin, Ireland; Dilip.Rai@teagasc.ie

**Keywords:** germination, lentil, GABA, antioxidant activity, glycemic index, response surface methodology (RSM)

## Abstract

Germination is an efficient and natural strategy that allows the modification of the nutritional value and the nutraceutical properties of seeds, enabling one to tailor the process according to its final use. This study aimed at optimization of germination conditions to produce novel lentil flours with improved nutritional and functional features. Response Surface Methodology (RSM) was applied to model the effect of temperature (15–27 °C) and time (1–5 days) on different nutritional and quality parameters of lentil flours including proximate composition, content and profile of fatty acids, content of phytic acid, ascorbic acid and γ-aminobutyric acid (GABA), content and profile of phenolic compounds, antioxidant activity, expected glycemic index (GI) and color during germination. As shown by RSM polynomial models, sprouting promoted the reduction of phytic acid content and enhanced the levels of ascorbic acid, GABA, insoluble phenolic compounds, antioxidant activity and expected GI, and modified the color of the resultant lentil flours. RSM optimization of germination temperature and time using desirability function revealed that the optimal process conditions to maximize the nutritional, bioactive and quality properties of sprouted lentil flours were 21 °C for 3.5 days.

## 1. Introduction

Malnutrition and unbalanced nutrition due to poor dietary choices are among the main factors that affect pathologies such as diabetes, obesity and/or anemia, with a major impact on infant and elderly populations, in which more than a third of total illnesses in the world are attributed to diet. In this sense, worldwide and especially European citizens are becoming increasingly conscious of healthy eating habits and dietary requirements [1]. In fact, there is an important interest of consumers for food products with clean labels, functional claims, natural sugars, and nutraceutical compounds [1]. The global pandemic caused by COVID-19 has contributed to an increase in these demands from consumers, and set opportunities for food manufacturers to redesign their marketing strategy related to healthy products. Therefore, the scientific literature has pointed to nutraceuticals and functional ingredients as a frontline protection to boost the immune system in the prevention of viral infections, such as COVID-19, in the population, especially young people, as adjunctive therapies [2,3]. Different authors have reported that these benefits can be associated with vitamins (C, D, E), minerals (zinc), melatonin, GABA, and other phytochemicals and functional ingredients, contributing to the disease prevention or control [1,2].

Natural ingredients provide benefits associated with key bioactive compounds, which can act by preventing or reducing the risk of developing different pathologies [4]. Grains, pulses and oilseeds are important sources of these compounds. Pulses, in addition to their high phytochemical content, provide a wide range of nutrients [5]. Unlike other plants, the *Leguminosae* family has the ability to contribute to biological nitrogen fixation to the soil and, therefore, make an important contribution to the achievement of many of the Sustainable Development Goals of the 2030 Agenda [6].

Despite their high nutritional value and the fact that they have been an essential part of the human diet for centuries, legume consumption is usually under-appreciated. Among pulses, lentils (*Lens culinaris*) have emerged as an interesting source of minerals such as iron, zinc, potassium magnesium, selenium, folate, thiamine, niacin and vitamin B_6_ and macronutrients such as slow digestible starch, protein and fiber [7]. A serving of 70 g of lentils covers 32% of the daily recommended intake of thiamine for adults, according to Annex XIII of Regulation 1169/2011 on food information to the consumer. In addition, consumer’s interest toward meat-based proteins is on the decline in different regions of the world due to growing awareness pertaining to the benefits of flexitarism, which will drive new opportunities for using lentils and other pulses.

Lentils are also rich in antioxidants, mostly associated with polyphenolic compounds, including flavonols, flavones, flavan-3-ols, proanthocyanidins, anthocyanidins, hydroxybenzoic, hydroxycinnamic acids, and isoflavones [8]. Different studies have shown that the consumption of lentil is associated with the reduction of diseases such as diabetes, obesity, cancer and other cardiovascular pathologies due to its polyphenol composition, which has been shown to possess antioxidant, anti-inflammatory and nephroprotective effects [8]. For instance, lentil flavonoids have been found to control α-glucosidase and lipase enzymes, resulting in potential postprandial blood glucose control [9]. This scientific evidence suggests that lentils might be a promising functional dietary ingredient.

Lentils are used in the form of whole seed, dehulled split grain and flour [10], which gives an edge to pulse flours over traditional cereal flours. The pulse market is increasing worldwide. Inclusion of lentil flour in food products may enhance their nutritional and health attributes [11], particularly of gluten-free foods, which often have an unbalanced nutritional composition [12].

Germination, also known as sprouting, is an emerging bioprocess to tailor and improve nutritional and bioactive properties of grains in a natural way [13,14,15]. Germination is induced by rehydration of the seed, which increases the metabolic pathways increasing the content and bioaccessibility of many nutrients and the production of secondary metabolites, many of them with antioxidant and other bioactive properties associated with health and well-being [16,17].

Although lentils have interesting nutritional value, they also contain anti-nutritional compounds such as trypsin inhibitors, phytic acid and tannins, which can interfere in protein and carbohydrate adsorption [18]. Germination can significantly diminish antinutrients, increasing the protein, carbohydrate and fat digestibility [19]. However, the germination process has a negative effect on the techno-functional attributes of the resultant flours, since the major soluble carbohydrate is in the dried seed. Breakdown of sucrose carbohydrates during sprouting leads to a marked increase in the production of glucose, whilst the increments of maltooligosaccharides accompanies the breakdown of starch. Maltotriose was found to constitute the greatest portion of the oligosaccharides during the germination.

The aim of this research was optimizing conditions applied during the germination process in order to develop a nutritious and highly antioxidant flour, suitable as a nutraceutical ingredient for the food industry, while maintaining its technological properties necessary to be integrated in food products. Furthermore, providing alternative uses of this commodity may help to promote the consumption of lentils in the Mediterranean area, where it is usually consumed in traditional dishes with unsprouted lentil seeds.

## 2. Materials and Methods

### 2.1. Chemicals

α-Amylase from porcine pancreas (EC 3.2.1.1), 2,2’-Azinobis 3-ethylbenzothiazoline-6-sulfonic acid (ABTS^•+^), 2,2’-diazobis-(2-aminodinopropane)-dihydrochloride (AAPH), 2,2-diphenyl-1-picrylhydrazyl (DPPH), Folin–Ciocalteu (FC) reagent, gallic acid (GA), 6-hydroxy-2,5,7,8-tetramethyl-2-carboxylic acid (Trolox), and sodium carboxy methyl cellulose (CMC) were obtained from Sigma-Aldrich, Co. (St. Louis, MO, USA).

The chromatographic standards: luteolin, luteoilin-4’*O*-glucoside, luteolin-7-*O*-glucoside, catechin, epicatechin, kaempferol-3-*O*-rutinoside, quercetin-3-*O*-glucoside, gallic acid, 2,3-dihydroxybenzoic acid, quinic, 4-hydroxybenzoic, p-coumaric, ferulic, and apigenin were obtained from Merck (formerly Sigma Aldrich, Arklow, Co., Wicklow, Ireland).

Amyloglucosidase (EC 3.2.1.3), glucose oxidase-peroxidase (GOPOD) and 1.3:1.4 mixed-linkage β-glucan kits were provided by Megazyme International Ireland (Wicklow, Ireland).

### 2.2. Lentil Seeds

*Lens culinaris* variety Pardina, derived from local ecotypes, adapted to the growing conditions in the Castilla y León (Spain) from the 2018–2019 campaign. Whole lentil grains from this variety were stored at 12–15 °C.

### 2.3. Germination Process

Twenty grams of lentil seeds were soaked in 100 ppm sodium hypochlorite (food grade) at a 1:5 ratio (*w*/*v*) for 30 min and then washed with tap water until they reached neutral pH. Afterwards, seeds were hydrated in tap water (at the same ratio) at room temperature for 6 h. Water was removed and seeds were spread across moist filter paper over a steel grid, which was placed in plastic trays containing sterile tap water. Seeds were covered by a filter paper and introduced in a germination cabinet (model MLR-350, Sanyo, Osaka, Japan) at >90% air humidity by capillarity. Germination was carried out in darkness at temperatures of 15–27 °C for 1–5 days, conditions chosen according to the response surface model (Table 1).

The germination process was replicated for each germination condition. The sprouted seeds were freeze-dried (LyoQuest, Telstar, Barcelona, Spain), grinded (Fidibus Medium, Komo, Penningberg, Austria) and fine-milled in a Cyclotec mill (Model CT 193, Foss, Hilleroed, Denmark) to a particle size < 0.5 mm. After this, fine powder was stored under vacuum at 15 °C until further analysis. Unsprouted lentil grains were similarly grinded and milled and the resultant flour was used as control sample.

### 2.4. Experimental Design

Response surface methodology (RSM), introduced by Box and Wilson [20], is a collection of mathematical and statistical techniques that can be used to optimize processes when the levels of one or more quantitative factors and their interactions may impact on the desired response. RSM allows one to predict the responses in conditions within the experimental domain where experiments were not conducted.

RSM was applied to study the effect of germination conditions (time and temperature) on different nutritional and bioactive parameters of germinated lentil. A central composite design (CCD) was employed with a total of 11 experimental points and three center points and “star” points to estimate curvature. The order of the experiments was randomly selected to avoid the effects of lurking variables (Table 1). The combination of the two factors (germination temperature and time) studied in the response surface designed and its optimization was based on the +1 and −1 variable level. Nutritional and bioactive parameters were selected as dependent variables. The ungerminated seed was used as control reference.

### 2.5. Proximate Compositon (PC) and Fatty Acid Profile (FAP)

Total protein content was determined by the Dumas method, AOAC method 990.03 [21], in an elemental analyser. A conversion factor of 5.7 was used to calculate protein content from nitrogen values. Total fat content was determined using dried samples extracted with petroleum ether (BP 40–60 °C) over 4 h in a Soxtec fat extracting unit (AOAC 2005, method 2003.05) [21]. Carbohydrates were estimated by difference. Total dietary fibre (TDF) content was evaluated using a kit provided by Sigma (TDF100A-1KT, St. Louis, MO, USA), in accordance with manufacturer’s instructions. Phytic acid and total phosphorus content were determined using a kit from Megazyme (K-PHYT, Bray, Ireland). All parameters were evaluated in duplicate. Proximal composition analysis was expressed in g 100 g^−1^ of dry matter (d.m.). All the analyses carried out in fatty acid composition was determined in the lipid-containing chloroform phase, which was separated and evaporated. After that, the sample was dissolved in 1 mL of hexane and methylated by the addition of 0.5 M methanolic KOH (100 μL) and incubated for 10 min at room temperature. An analysis of fatty acid methyl esters (FAME) in the upper layer obtained was performed on a gas chromatograph (Agilent 7890A, Agilent Technologies, Wilmington, CA, USA) coupled with a flame ionization detector. FAME’s were identified by comparison of retention times with those of the standard (37 FAME’s mix, Supelco, Sigma-Aldrich, Wicklow, Ireland). Results of FAP were expressed as g 100 g^−1^ of total fatty acids. All the analyses were carried out in duplicate.

### 2.6. Determinion of Malondialdehyde (MDA)

Lipid oxidation was measured through the analysis of malondialdehyde (MDA) using the method described by Vyncke [22]. Analyses were carried out in duplicate. Results were expressed as mg of MDA per kg of flour.

### 2.7. Content of Ascorbic Acid

Ascorbic acid was determined in lentil flour samples by homogenization of the sample with 50 mL of oxalic acid (2 g 100 g^−1^) and titration with 2,6-dichlorolindophenol (0.25 g L^−1^) using a potentiometric titrator (877 Tritino Plus, Metrohm, Herisau, Switzerland). Analyses were carried out in duplicate. The results were expressed as mg ascorbic acid 100 g^−1^ d.m.

### 2.8. Content of γ-Aminobutyric Acid (GABA)

GABA was determined in water extracts obtained from raw and sprouted lentil flour samples as previously reported [23]. Briefly, an aliquot (50 μg) of extract was combined with an allyl-L-glycine solution (10 μL, internal standard) and derivatized with phenyl isothiocyanate (30 μL). Then, the resultant extract was dried in a vacuum concentrator and the sample was solubilized in 0.1 M ammonium acetate, pH 6.5 for GABA quantification, which was performed using High-performance liquid chromatography (HPLC) coupled to a diode array detector [23]. Analyses were carried out in duplicate. Results were expressed as mg 100 g^−1^ d.m.

### 2.9. Content of Total Phenolic Compounds Determined by the Folin-Ciocalteu Method

Content of total phenolic compounds (TPS) were quantified in methanolic extracts obtained from control and sprouted lentil flours. Briefly, 1 g of sample was combined with 10 mL of methanol:water (1:1, *v*/*v*; acidified to pH 2 with 0.1 M HCl) and stirred (250 rpm, 25 °C, 1 h) in an orbital shaker. After centrifugation (25 °C, 3800× *g*, 10 min), the supernatant was collected, filtered through Whatman paper n° 1, and made into a final volume of 25 mL with the extracting solvent. Extracts were stored at −80 °C until use. s were determined using the Folin-Ciocalteu method [24]. Gallic acid (GA) was used as standard. Analyses were carried out in duplicate. The final results were corrected for moisture content and expressed as µmol GA equivalents (GA Eq) g^−1^ d.m.

### 2.10. Determination of Free and Bound Phenolic Compounds Using HPLC-Q-TOF-MS/MS and UPLC-TQD

Free phenolic compounds were extracted as reported in Section 2.8. The precipitates obtained after free phenolic extraction were subjected to a two-step hydrolysis at alkaline (10 M NaOH) and acidic (pH 2, HCl) conditions for recovering the bound (insoluble) fraction, as reported by Mattila et al. [25]. Soluble and insoluble phenolic extracts were filtered through a 0.22 µm filter, freeze-dried and stored at −20 °C until analysis.

Soluble and insoluble phenolic compounds were qualitatively and quantitatively analyzed by adapting the Liquid chromatography-mass spectrometry (LC-MS/MS) methods described previously by Gangopadhyay et al. [26]. Characterization and identification of the phenolic compounds was performed using Q-ToF mass spectrometry coupled to Alliance HPLC 2695 (Waters Corporation, Milford, MA, USA). The equipment was controlled using the Masslynx software v. 4.1 (Waters Corporation, Milford, MA, USA). The samples (5 μL) were injected on to an Atlantis T3 column C18 column (3 μm, 100 × 2.1 mm; Waters, Milford, MA, USA). The components were eluted using 0.1% formic acid in water (mobile phase A) and 0.1% formic acid in acetonitrile (mobile phase B) at a flow rate of 0.3 mL/min using the gradient program from 10% B to 90% B in 25 min. Mass spectrometry analysis was performed using a negative ion mode for a mass scan range from *m*/*z* 70 to 1000. The Q-ToF mass spectrometry analysis parameters used were: capillary and cone voltages were set at 3 kV and 30 V, respectively; sample cone and desolvation gas temperatures were set at 150 °C and 350 °C, respectively; collision-induced dissociation (CID) of the analytes was performed using argon at 12–20 eV. The phenolic compounds were identified according to their accurate mass measurements, CID fragmentation patterns, literature and using available commercial standards.

Quantification of the phenolic compounds was performed on a ultra performance liquid chromatography-tandem mass spectrometer (UPLC-MS/MS) (UPLC-TQD, Waters Corp., Milford, MA, USA), where the separation of the analytes was achieved on an Acquity UPLC HSS T3 column (100 × 2.1 mm, 1.8 µm) using the binary solvents A and B as above with an increasing organic solvent gradient from 2% B to 98% B at a flow rate of 0.5 mL min^−1^ for 9.5 min. The column temperature was set at 50 °C, while the samples were kept at 4 °C. Detection and quantification of the phenolic compounds in the UPLC-TQD were performed in multiple reaction monitoring (MRM) mode by analyzing at least two transitions per compound. The cone voltages and collision energies were optimized for MRM transition ions while using IntelliStartTM software (Masslynx 4.1, Waters Corp., Milford, MA, USA). Analyses were carried out in triplicate extracts and target compounds were quantified using standard calibration curves of concentrations that ranged from 0.02 µg mL^−1^ to 10 µg mL^−1^. The results were expressed as µg g^−1^ of sample.

### 2.11. Total Antioxidant Capacity (TAC)

The antioxidant activity was measured in extracts (Section 2.9) previously obtained using Ferric Reducing Ability Potential (FRAP), Oxygen Radical Absorbance Capacity (ORAC), 2,2-Diphenyl-1-picrylhydrazyl radical (DPPH^•^) and 2,2’-Azinobis-(3-ethylbenzothiazoline-6-sulfonate (ABTS^•+^) assays. In addition, DPPH^•^ and ABTS^•+^·modified methods were applied on solid samples without previous extraction (Q-DPPH^•^and Q-ABTS^•+^), in order to evaluate the total antioxidant activity of the samples. Samples were evaluated in duplicate.

#### 2.11.1. Ferric Reducing Antioxidant Power (FRAP)

FRAP assay was performed following the protocol reported by Benzie et al. [27]. Absorbance at 700 nm was recorded. FeSO_4_·7H_2_O (4.0–2.2 mM) was used to evaluate Fe reduced in the assay. Analyses were carried out in duplicate. The results were expressed as mmol Fe Eq. reduced 100 g^−1^ sample.

#### 2.11.2. Oxygen Radical Absorbance Capacity (ORAC)

This procedure was based on a previously reported method by Ou et al. [28], with modifications. Standard curve of Trolox (7.5–240 mM) and samples were diluted in phosphate buffer (10 mM, pH 7.4). Fluorescence was monitored over 150 min with a microplate reader (Fluostar Omega, BMG, Ortenberg, Germany), using 485 nm excitation and 520 nm emission filters. Analyses were carried out in duplicate. Results were calculated using the areas under the fluorescein decay curves, between the blank and the sample, and expressed as µmol Trolox Eq. 100 g^−1^ sample.

#### 2.11.3. Radical Scavenging Activity (DPPH^•^) and Quencher Radical Scavenging Activity (Q-DPPH·)

The extract-based DPPH assay was carried out as described by Brand-Williams et al. [29], with modifications. A 120 µM DPPH^•^ working solution was prepared in pure methanol. In a 96-well microplate, a volume of 25 µL of extracts was mixed with 100 µL of milliQ water and 125 µL of DPPH^•^ working solution. The decay in absorbance at 525 nm was recorded over 30 min with a microplate reader (Fluostar Omega, BMG, Ortenberg, Germany). Different Trolox concentrations (7.5–240 µM) were used to perform the calibration curve. Analyses were carried out in duplicate. Results were expressed as µmol Trolox Eq. 100 g^−1^ of flour.

The solid sample-based Q-DPPH^•^ method was assayed according to the procedure by Serpen et al. [30], with modifications. Ten milligrams of powdered solid samples (<300 µm) were mixed with 30 mL of DPPH^•^ working solution (60 µM) prepared in methanol. After incubation for 30 min at 700 rpm (Thermomixer Compact, Eppendorf, AG, Hamburg, Germany), samples were centrifuged at 14,000× *g* for 2 min and the absorbance measured at 515 nm. Analyses were carried out in duplicate. Results were expressed as µmol Trolox Eq. 100 g^−1^ of flour.

#### 2.11.4. Radical Cation Scavenging Activity (ABTS^•+^)

ABTS^•+^ was evaluated following the method first described by Miller and Rice-Evans [31], as modified by Martin-Diana et al. [32]. The absorbance was measured at 730 nm. Analyses were carried out in duplicate. Results were expressed as µmol Trolox Eq. 100 g^−1^ of flour.

### 2.12. Glycemic Index (GI)

Determination of GI was performed by a two-step’s procedure. Firstly, the content of total starch was quantified using the total starch assay kit of Megazyme, Bray, Ireland (K-TSTA 08/16). Then, the in vitro starch hydrolysis rate was determined according to Gularte and Rosell [33], with slight modifications. Samples containing 50 mg of available starch were dissolved in tris-maleate buffer (0.1 M, pH = 6, 2 mL) and combined with 2 mL of an enzymatic solution containing porcine pancreatic α-amylase (460 U mL^−1^) and amyloglucosidase (6.6 U mL^−1^). Aliquots were taken during the incubation period (150 min) and immersed in boiling water for 5 min to inactivate enzymatic activity present in the aliquots. After cooling the sample in ice, 150 µL of absolute ethanol was added and the sample was centrifuged (4 °C, 10,000× *g* for 5 min). The pellet was washed with 150 µL ethanol:water (1:1, *v*/*v*) and the supernatants were pooled together and stored at 4 °C for the subsequent colorimetric analysis of reducing sugars using the GOPOD kit (Megazyme, Bray, Ireland). Expected GI values were calculated from hydrolysis index (HI) values, as proposed by Granfeldt [34].

### 2.13. Colorimetric Characterization of Germinated Lentil Flours

Color was measured using a colorimeter (Minolta CM-2002, Osaka, Japan) with D65 as illuminant and 45/0 sensor. The instrument was calibrated with a white tile and light trap, following manufacturer instructions. The values obtained in the space CIE L*a*b* (*CIELAB*) were converted to Hue (arctan b*/a*) and Chroma (a*^2^ + b*^2^)^1/2^ based in the CIELab (L*, a* and b*).

### 2.14. Statistical Analysis

A CCD was used to study the quadratic response surfaces and to obtain second order polynomial equations for each response variable. The response variable (Y) was described by the following regression model (Equation (1)):

(1) $$Y=\beta_{0}+\Sigma_{i=1}^{2}\beta_{i}X_{i1}+\Sigma_{i=1}^{2}\beta_{ii}X_{i1}^{2}+\beta_{ij}X_{1}X_{2}$$

where *β*_0_ is a constant coefficient, *β_i_*_,_ *β_ii_* and *β_ij_* are the linear, quadratic, and the interaction coefficients, respectively. *X*_1_ and *X*_2_ are the factors, germination temperature and time, respectively. ANOVA was applied to determine the significance of the second order models. The latter was considered satisfactory when the regression was significant, and a non-significant lack of fit was obtained for the selected confidence level (α = 0.05). The coefficient of determination (*R*^2^) and the adjusted coefficient of determination were also evaluated to confirm that variation of the data was mainly explained by the model. Multi-response optimization was performed using the desirability function, which enables one to find the conditions that maximize the parameters with higher weight or importance in the optimization. For the desirability function, 12 parameters were considered in order to obtain the conditions (temperature and time) where phenols, antioxidant activity (ABTS^•+^, ORAC, FRAP and Q-DPPH^•^), ascorbic acid, protein, polyunsaturated fatty acids and fiber were maximized and GI was minimized). All statistical analyses were performed using Statgraphics Centurion XVI^®^ (StatPoint Technologies, Inc., Warrenton, VA, USA).

## 3. Results and Discussion

### 3.1. RSM Modeling of Germination Conditions and Their Impact on Sprouted Lentil Flours

The understanding of the effect of germination conditions on nutritional, bioactive and techno-functional properties of sprouted lentil flours is essential for designing novel food products derived from sprouted flours with high-added value. Therefore, experimental data obtained after running the 11 experiments indicated by RSM were fitted to quadratic second-order polynomial models. The mathematical models for each response variable studied are shown in Table 2. All of the models were statistically significant (*p* ≤ 0.05) and showed a non-significant (*p* > 0.05) lack of fit, revealing their suitability for modeling the experimental data. Only the significant (*p* ≤ 0.05) regression coefficients were included in the quadratic equations. The value of the coefficient of determination (R^2^) indicates the suitability of the RSM models to explain the variability of experimental results. Three-dimensional response surface plots explain the interaction between germination time and temperature and the relationship between germination conditions and the response variables studied.

### 3.2. Proximate Composition of Sprouted Lentil Flours

**Figure 1:**
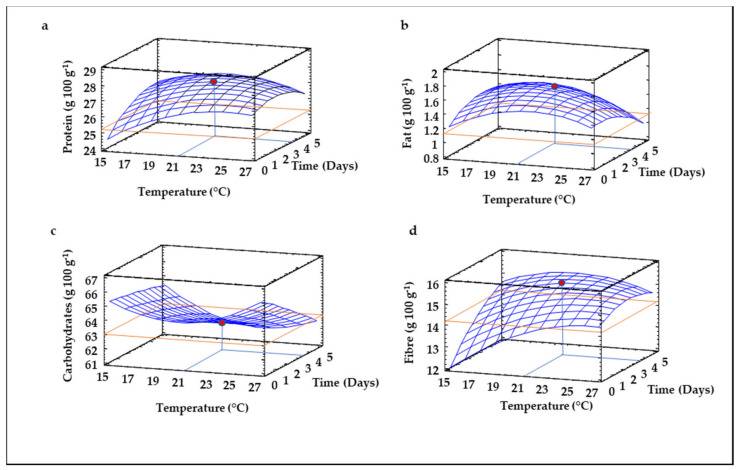
Three-dimensional Response Surface Plots for the content of total protein (**a**), fat (**b**) carbohydrates (**c**), and fiber (**d**) as a function of the interaction between germination temperature and time. The red line corresponds with the control (ungerminated seed) and the red point with the value for optimum conditions (21 °C and 3.5 days).

Proximate composition was analyzed in raw and germinated lentil flours in order to evaluate the effect of germination temperature and time on the contents of protein, fat, carbohydrates, fiber, moisture and ash. Figure 1 depicts the response surface plots obtained for these response variables.

Control flour (unsprouted lentil flour) showed a protein content of 25.16 ± 0.13 g 100 g^−1^, which was similar to that reported in lentil flour by Khaur and Sandhu [35]. As evidenced by the RSM model obtained, both germination temperature and time exerted mainly linear effects, although the quadratic effect and the interaction between both factors was also significant (*p* ≤ 0.05). The model explained 69% of the variability of experimental data, in view of the R^2^ value obtained (Table 2). The predictive RSM plot (Figure 1a) revealed that increases in temperature from 15 °C to 23 °C significantly favored (*p* ≤ 0.05) the increase in protein in sprouted lentil flour, independently of the germination time. Time produced similar effects on protein content since it increased as germination time augmented until 3–4 days, decreasing at longer times. The maximum protein value in sprouted lentil flour was predicted at 22 °C and 3 days (Table 2). These results agree with studies performed by other authors where an enhancement in protein levels was observed during lentil germination beyond 3 days at 20–25 °C [36,37,38]. The high amino acid biosynthetic activity that takes place during germination and the synthesis of enzymes in sprouting seeds [38,39] may be responsible for the increased protein levels in sprouted lentil flour. Furthermore, the losses of dry weight in the form of carbohydrates through respiration can also contribute to the increases in protein content of germinated flours [40].

Germination temperature and time showed a linear impact on fat levels of sprouted lentil flour, but the mathematical model obtained showed a poor agreement between experimental and predicted data, considering the low R^2^ value (0.34, data not shown). As depicted in Figure 1b, germination caused a decrease in fat content during germination, with the lowest fat values at higher temperatures and longer times, in agreement with other authors, who observed reductions of lipid content from 17 to 68% in lentil germinated for 4–6 days at 25 °C [38,41]. The activation during sprouting of endogenous lipolytic enzymes that hydrolyze triacylglycerol to release free fatty acids, which are oxidized to produce energy for seedling development [38], is mainly responsible for the reduction in fat content in germinated lentil flour. There is a strong interest in the production of flours with low fat content, with the objective to avoid rancidity and extend their shelf-life. In this context, longer germination times would enhance fat hydrolysis, where 27 °C and 5 days are the optimal germination conditions to achieve this purpose (Table 2, Figure 1b).

Lentil seed showed a content of carbohydrates of 62.96 ± 0.39 g 100 g^−1^ response variables, levels close to that observed by Atudorei et al. [41], who found a value of 60 g 100 g^−1^ in non-sprouted lentil. Germination temperature and time performed quadratic and linear and interaction effects on the levels of carbohydrates (Table 2). The reliability of the RSM model obtained was high (R^2^ = 0.72, Table 2). The impact of temperature did not show a clear trend, since both low and high temperatures caused the highest carbohydrate increment, while medium temperatures were related with the lowest carbohydrate levels. In contrast, longer times reduced the content of carbohydrates in sprouted lentil flour, especially at higher temperatures (Figure 1c), findings in agreement with those reported in lentil germinated for 1–4 days [41,42]. The activation of a wide range of hydrolytic enzymes, including α-amylase, glucosidase, and dextranase existing in the aleurone layers, and β-amylase present in the endosperm that hydrolyze starch to produce energy [43] may be involved in the reduction of carbohydrate content during the germination.

Fiber was also influenced by linear, quadratic and interaction effects of both germination temperature and time, being the second-order equation obtained by RSM suitable to explain the variation in experimental data, considering the R^2^ value (0.63, Table 2). The three-dimensional response surface plots revealed that fiber content increased as temperature and time increased, but higher temperature combined with longer time negatively affected fiber levels of germinated lentil flour (Figure 1d). Combinations of 21 °C and 4.5 days of germination caused the highest fiber increase in germinated lentil flour. The results regarding the influence of germination on dietary fiber content of legumes are not conclusive. Several studies conducted with other legumes (*Vigna unguiculata* L., *Canavalia ensiformis, Stizolobium niveum* L. *and Lablab purpureus* L.), cowpea and lentil revealed that germination at 25 °C for 96 h enhanced both insoluble and soluble dietary fiber fractions, as well as insoluble/soluble dietary fiber ratio [42,44,45]. Contrarily, a decrease in total dietary fiber due to the decrease in the insoluble fraction in lentil and wrinkle brown lentil was observed by other authors [46,47], results attributed by the authors to the reduction in hemicellulose content during germination and cell-wall degradation, probably because the decrease in soluble dietary fiber during lentil germination occurred to a lower extent than insoluble fiber content, resulting in an overall enhancement in total dietary fiber, as previously observed in germinated wheat [48,49,50].

### 3.3. Fatty Acid of Sprouted Lentil Flours

**Figure 2:**
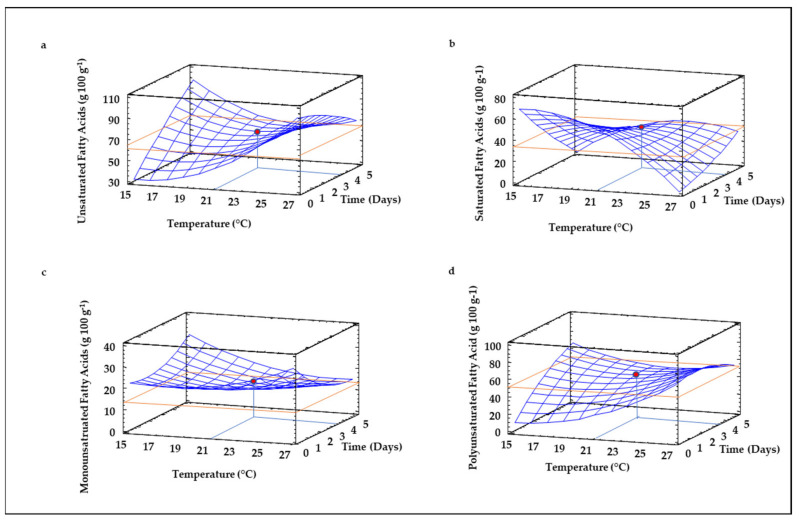
Three-dimensional Response Surface Plots for relative percentage of unsaturated fatty acids (**a**), saturated fatty acids (**b**), monounsaturated fatty acids (**c**) and polyunsaturated fatty acids (**d**) as a function of the interaction between germination temperature and time. The red line corresponds with the control (ungerminated seed) and the red point with the value for optimum conditions (21 °C and 3.5 days).

The fatty acid composition was evaluated due to its importance on the quality during the shelf-life of the resultant flours, since double bonds of unsaturated fatty acids are highly reactive and may produce rancidity and undesirable flavors during storage [18,51]. RSM models revealed that only linear terms of germination time and temperature showed a significant (*p* ≤ 0.05) influence on the levels of saturated and unsaturated (mono- and polyunsaturated) fatty acids. However, the goodness-of-fit of the equations obtained was low (R^2^ ≤ 0.29) (Table 2), indicating that other germination factors different from time and temperature might be involved in the fatty acid composition of sprouted lentils. Low germination temperatures (15–20 °C) brought about a lower increase in unsaturated fatty acids compared to saturated fatty acids, which may be explained by the lower melting point and increased mobilization of the former fatty acids. Over sprouting time, the balance between saturated and unsaturated fatty acids decreased (Figure 2a,b). At higher temperatures (23–27 °C), no changes in the balance of saturated and unsaturated fatty acids is observed, suggesting a more stable lipid metabolism and non-adaptation. Among unsaturated fatty acids, monounsaturated fatty acids did not remarkably change during germination (Figure 2c), while polyunsaturated fatty acids increased at low-medium temperatures as the germination time increased (Figure 2d).

The evaluation of fatty acid composition of non- and germinated lentil flours (data not shown) showed that the linoleic acid was the predominant fatty acid (52.54% of total FFA, C18:2 (n6), followed by palmitic acid (29.75%, C16:0), oleic acid (13.37% C18:1 (n9) and stearic acid (4.34% of total FFA, C18:0), values that agreed with other authors [18,51]. The linoleic acid percentage suffered the highest reduction during the germination process (data not shown, from 83% to 37%), data also in agreement with values reported by Pal et al. [18].

### 3.4. Content of Malondialdehyde (MDA) of Sprouted Lentil Flours

**Figure 3:**
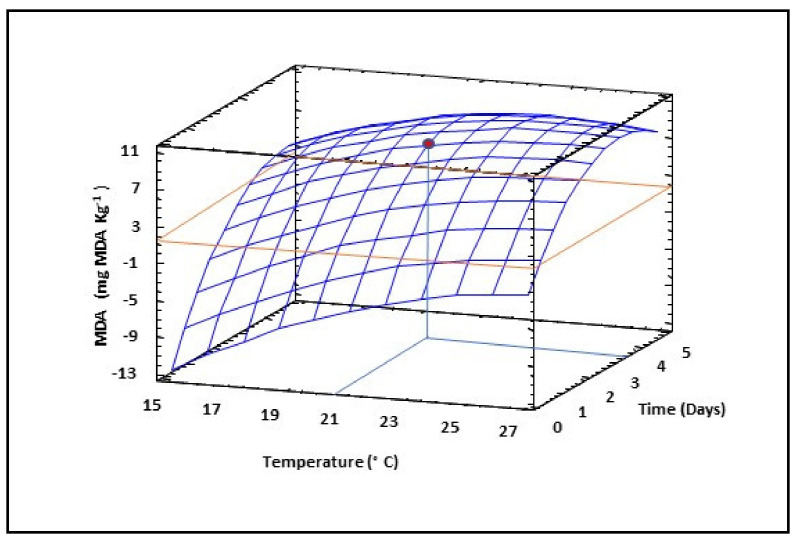
Three-dimensional response surface plots for the content of total malondialdehyde (MDA) as a function of the interaction between germination temperature and time. The red line corresponds with the control (ungerminated seed) and the red point with the value for optimum conditions (21 °C and 3.5 days).

Thiobarbituric acid reactive substances were used as an indicator of lipid peroxidation and the extent of oxidative state of lentil flours. The assay study the reaction of lipid peroxidation products, malondialdehyde (MDA), with thiobarbituric acid (TBA), which leads to the formation of MDA-TBA, known as thiobarbituric acid reactive substances (TBARS). Therefore, measurement of MDA content can accurately evaluate the fat oxidation degree. Non-sprouted lentil seeds showed levels of MDA of 0.17 ± 0.03 mg kg^−1^. The quadratic model obtained indicated that TBARS were affected by quadratic, linear and interaction effects of germination temperature and time, polynomial model explained 96% of the variability of MDA (Table 2). Increases in germination temperature and time negatively affected the quality of sprouted lentil flours since they enhanced the increase in MDA (Figure 3). Combinations of germination temperatures higher than 25 °C with for longer than 3.7 days should not be applied to lentils in order to obtain high-quality sprouted flours.

### 3.5. Phytic Acid Content of Sprouted Lentil Flours

**Figure 4:**
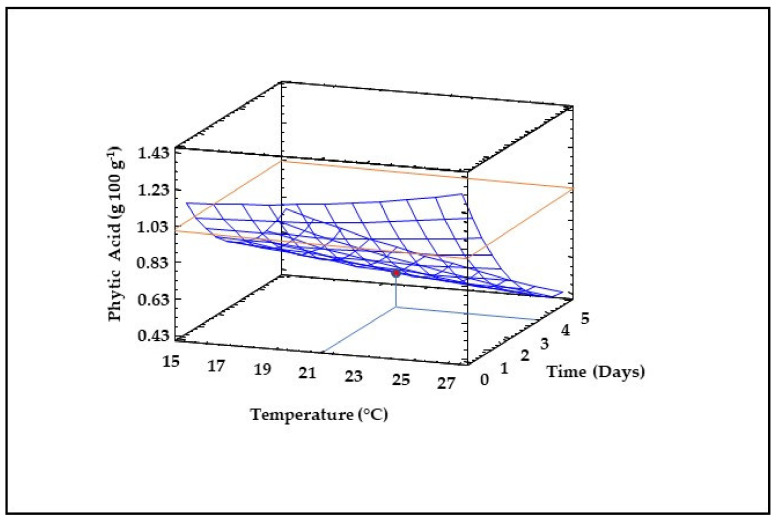
Three-dimensional response surface plots for phytic acid content as a function of the interaction between germination temperature and time. The red line corresponds with the control (ungerminated seed) and the red point with the value for optimum conditions (21 °C and 3.5 days).

The reduction in the levels of antinutrients is an interesting strategy to produce flours with higher nutritive value. Phytic acid is one of the most important antinutrients in lentils, since it binds dietary minerals, proteins and starch, consequently reducing their bioavailability [52]. Control flour showed a phytic acid content of 0.9 g 100 g^−1^ (data not shown), close to those reported by Montemurro et al. [52] and higher than values reported by Pal et al. [18] in sprouted lentils (0.25–0.38 g 100 g^−1^). Both germination temperature and time exerted quadratic, linear and interaction effects on phytic acid levels (Table 2) and the model obtained was highly suitable for explaining the variations in the experimental data as a function of germination temperature and time (R^2^ = 0.81, Table 2). Germination time caused a more pronounced impact than temperature on the content of this antinutritional compound, which decreased as germination time increased, especially at higher temperatures (Figure 4). In agreement with our results, previous studies have shown reduced phytic acid content in sprouted cereals and legumes [18,36,42,52]. The results of the present study suggest that activation of endogenous phytases, enzymes that hydrolyze phytic acid to lower inositol phosphates and free phosphorus in sprouted lentils occurs at longer sprouting times, and reductions of 50% in the levels of this compound can be achieved at germination times of 5 days (Figure 4).

### 3.6. Ascorbic Acid Content of Sprouted Lentil Flours

**Figure 5:**
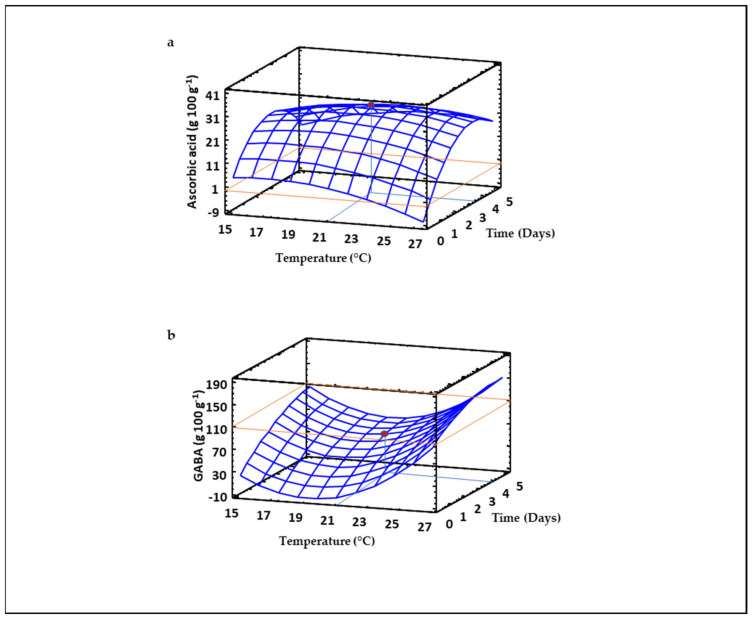
Three-dimensional response surface plots for the content of ascorbic acid (**a**) and GABA (**b**) as a function of the interaction between germination temperature and time. The red line corresponds with the control (ungerminated seed) and the red point with the value for optimum conditions (21 °C and 3.5 days).

The production of flours with high content of ascorbic acid is interesting not only from the nutritional point of view, but also for improving their stability over the shelf-life. The RSM model indicated that germination temperature and time exerted linear and quadratic effects on the content of this vitamin, with the interaction between both factors also being significant (*p* ≤ 0.05). However, the model explained less than 40% (Table 2) of the variability in the experimental data, pointing to the contribution of other factors in addition to germination temperature and time on ascorbic acid content. As shown in Figure 5a, germination time had a strong influence on ascorbic acid content in sprouted lentil, which increased as germination time augmented up to 3.5 days, while temperature did not exert a notable effect. Our results agree with those previously reported in different cultivars of mung bean and soybean [53,54], where germination up to 4 days maximized the content of ascorbic acid, decreasing at longer germination times. Since ascorbic acid is synthesized from glucose, mannose, and galactose [53], an increase in ascorbic acid can be partially due to the enzymatic hydrolysis of starch by amylases in sprouted lentil, which increases the bioavailability of glucose. The activation of L-galactono-γ-lactone dehydrogenase, which catalyzes the oxidation of L-galactono-1,4-lactone to ascorbic acid [55] may also contribute to the increase in ascorbic acid in sprouted lentil flours.

### 3.7. GABA Content of Sprouted Lentil Flours

The content of GABA was quantified in sprouted lentil flours due to their widely known health benefits and the recent food industry trend towards the production of GABA-enriched foods [56,57]. The RSM mathematical equation obtained for modelling GABA levels in sprouted lentil as a function of germination temperature and time (Table 2) revealed that the increase of this non-protein amino acid in lentil was affected by quadratic, linear and interaction terms of both germination temperature and time. The model obtained predicts 67% of the variability of GABA experimental data (Table 2). The tridimensional response surface plot (Figure 5b) illustrates the enhancement of GABA levels in sprouted lentil flour as the time and temperatures increased from 1 to 5 days and from 19 to 27 °C, respectively. These results support previous studies showing the positive impact of germination on the content of GABA in different grains [13,14,58,59,60]. GABA is synthesized by the decarboxylation of L-glutamic acid catalyzed by (GAD). Sprouted lentils are potential dietary sources of GABA due to their high protein and L-glutamic acid concentration [36]. Germination causes the partial hydrolysis of proteins, increasing the availability of free glutamic acid, which together with the activation of GAD [61] are responsible for GABA enhancement in sprouts obtained at higher temperatures and longer germination times. GABA has multiple health-promoting properties such as reduction of blood pressure, inhibition of tumor cell proliferation, stimulation of the immune system, improvement of brain function and antidiabetic effects [57]. Hence, germination at high temperatures and long times can be identified as an economic approach to produce functional sprouted lentil flours enriched in GABA.

### 3.8. Content of Total Phenolic Compounds (TPS) in Sprouted Lentil Flours

**Figure 6:**
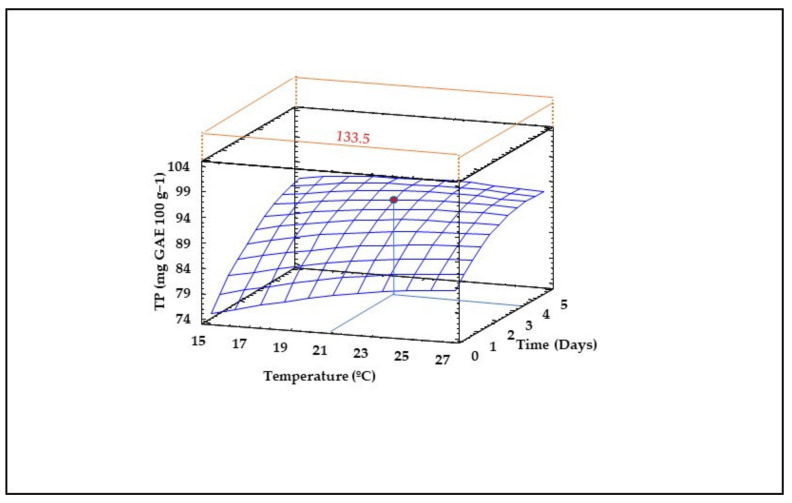
Three-dimensional response surface plots for Total Phenol (TP) as a function of the interaction between germination temperature and time. The red line corresponds with the control (ungerminated seed) and the red point with the value for optimum conditions (21 °C and 3.5 days).

RSM modelling revealed that both germination temperature and time exert significant quadratic, linear and interaction effects on TPS content of sprouted lentil flours (Table 2). However, the reliability of the model was low, in view of the poor R^2^ value obtained (R^2^ = 0.34). As shown in Figure 6, time had a stronger positive impact on TPS increase than temperature. The highest TPS content (~100 mg GA 100 g^−1^) in sprouted lentil flours predicted by the model occurs after germination at 21–25 °C and 5 days, levels that are more than 30% higher than those found in non-sprouted lentil flour (75 mg GA 100 g^−1^) (Figure 6).

The increased content of TP in sprouted lentil flours compared to the control may respond to the release of bound phenolic compounds during germination [13,15,62,63], and to a lesser extent, it may be due to the increase in phenylalanine ammonia lyase (PAL) activity, an enzyme involved in de novo biosynthesis of phenolic compounds [62]. The reduction observed in TPSs before germination compared to the control was due to the soaking treatment, which must be affected by the solubilization of some phenols during the germinative process since the seed maintained the humidity by capillarity, which can promote the loss of soluble compounds in the leech of the tray and accelerate the activation of enzymes such as polyphenol oxidase.

### 3.9. Profile of Free and Bound Phenolic Compounds in Sprouted Lentil Flours

**Figure 7:**
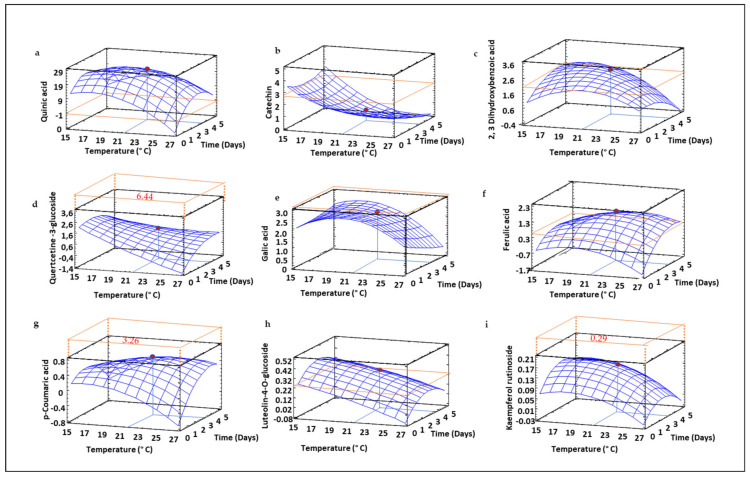
Three-dimensional response surface plots for the content (µg mg^−1^) of insoluble phenolic compounds determined using High-performance liquid chromatography (HPLC) coupled to a tandem quadrupole mass spectrometer: Quinic acid (**a**), Catechin (**b**), 2,3, Dihydrobenzoic acid (**c**), Quertcetin-3-glucoside (**d**), Gallic acid (**e**), Ferulic acid (**f**), p-Coumaric acid (**g**), Luteolin-4-O-glucoside (**h**), and Kaempferol rutinoside (**i**) as a function of the interaction between germination temperature and time. The red line corresponds with the control (ungerminated seed) and the red point with the value for optimum conditions (21 °C and 3.5 days).

Phenolic compounds in free and bound fractions of sprouted lentil flours were analyzed and quantified and the changes in their content due to germination temperature and time were modeled. The insoluble fraction (bound phenolic compounds) was mainly composed of catechin, 2,3 dihydroxybenzoic quercetin-3-glucoside, gallic acid, ferulic acid, *p*-coumaric acid, luteolin-4-*O*-glucoside and kaempferol rutinoside. The cyclic poliol (quinic acid) was also identified in this fraction. Moreover, in Figure 7, all of these compounds showed a germination time-dependent increase in sprouted lentil, with the exception of catechin and gallic (Figure 7b). A significant impact of temperature was also noticed, decreasing phenolic levels as the sprouting temperature increased. The maximum levels for most of the individual phenolic compounds identified were observed with use of moderate germination times (2.5 days) and temperatures (21–23 °C).

**Figure 8:**
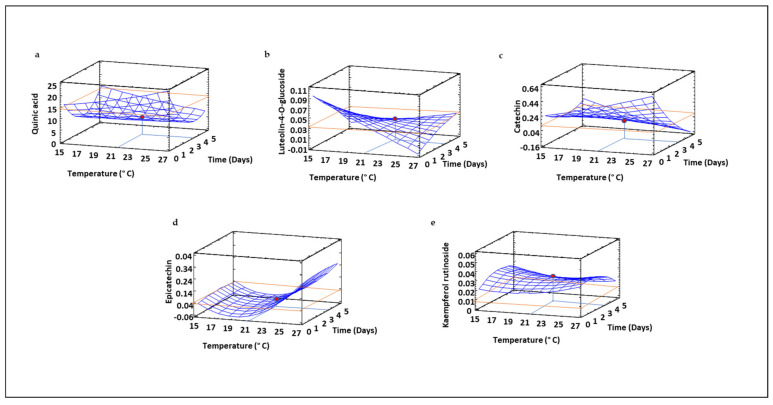
Three-dimensional response surface plots for the content (µg mg^−1^) of soluble phenolic (µg/mg) compounds determined using High-performance liquid chromatography (HPLC) coupled to a tandem quadrupole mass spectrometer: Quinic acid (**a**), Luteolin-4-*O*-glucoside (**b**), Catechin (**c**), Epicatechin (**d**), and Kaempferol rutinoside (**e**) as a function of the interaction between germination temperature and time. The red line corresponds with the control (ungerminated seed) and the red point with the value for optimum conditions (21 °C and 3.5 days).

Regarding the soluble fraction, the main phenolic compounds identified were quinic acid, luteolin-4-*O*-glucoside, catechin, epicatechin, and kaempferol (Figure 8), results that align with those observed by Zhang et al. [9], who also found catechin, epicatechin and kaempferol as the major soluble phenolics in a collection of 20 Canadian green and red lentil varieties.

Regarding soluble phenolics (Figure 8, Tables S1 and S2), luteolin, catechin, epicatechin and kaempferol were the main polyphenolic compounds determined. Zhang et al. [9] also found catechin, epicatechin and kaempferol to be the majority soluble phenolics in a collection of 20 Canadian unsprouted green and red lentil varieties.

The changes in the different soluble phenolics during germination showed different trends. In our study, the cyclic polyol quinic acid was also found in the soluble fraction of sprouted lentil flours. It is a compound that was reduced as the germination time increased up to 2.5 days, and then increased up to 5 days (Figure 8a). The behavior of quinic acid levels in the soluble fraction was the opposite of that observed in the insoluble one (Figure 7a), as it increased up to 2.5 days and then decreased over longer times. These results can be explained by the migration of this compound from the soluble fraction contained in cell vacuoles during germination to form novel cell wall structures [9]. The flavone luteolin-4-*O*-glucoside and the flavan-3-ol catechin exhibited a complex behavior, since the germination time and temperature exerted interactive effects on their content, increasing the content of luteolin-4-*O*-glucoside (Figure 8b) as the time increased at higher temperatures, while the content of catechin (Figure 8c) showed the opposite behavior. Epicatechin (Figure 8c) showed a different trend, with a clear temperature dependency, while time did not exhibit important effects on the content of this compound. In contrast, kaemperol rutinoside content depended mainly on germination time but temperature was not significantly affected. Epicatechin and quinic acid have been shown to reduce inflammation through NF-κB modulation [64,65,66]. In this regard, germination times of 2.5 days combined with high temperatures may improve the anti-inflammatory potential of sprouted lentil flour by increasing the content of both compounds in the soluble fraction.

It should be noticed that individual phenolic levels in insoluble and soluble fractions did not fully reflect the behavior of total phenolic compounds (Figure 6). An increase in phenolic levels during germination has been described as a defense reaction related to external stress [67]. In this work, RSM modelling revealed that temperature increases resulted in higher total TPS values, but this behavior was not observed in most of the individual phenolic compounds identified, probably due to the poor reliability of the polynomial equation obtained for TPS, as previously explained. However, it should be noticed that the change of phenolic profile due to the germination process and the increase of soluble forms of some individual phenolic compounds such as epicatechin is of a great interest in order to increase its bioavailability.

### 3.10. Antioxidant Activity of Sprouted Lentil Flours

**Figure 9:**
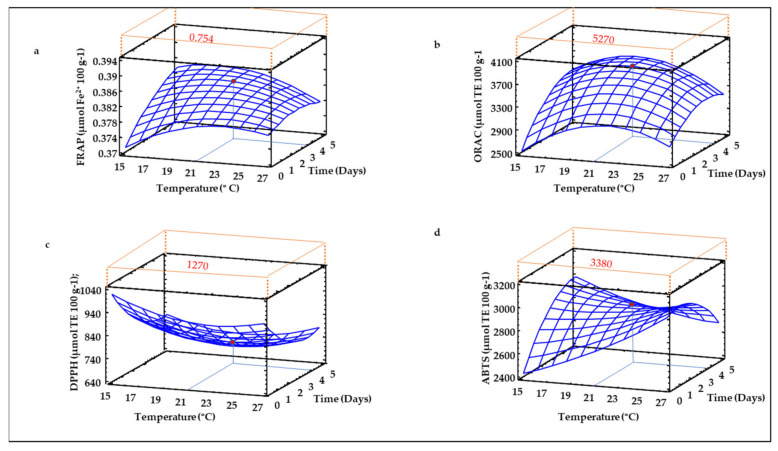
Three-dimensional response surface plots for antioxidant activity measured by FRAP (mmol Fe^2+^ 100 g^−1^) (**a**), ORAC, DPPH^•^, ABTS^•+^ (µmol Eq. Trolox 100 g^−1^) (**b**–**d**) as a function of the interaction between germination temperature and time. The red line corresponds with the control (ungerminated seed) and the red point with the value for optimum conditions (21 °C and 3.5 days).

The antioxidant activity of sprouted lentil flours was evaluated by different methods in order to have a more reliable knowledge of their total antioxidant potential (Figure 9). The modelling of the results obtained in the different antioxidant assays applied indicated that time exerts a positive effect on the antioxidant activity of sprouted lentil flour, although these results were not observed in the DPPH radical scavenging assay, where the antioxidant potential decreased as the germination time increased. These results agree with those reported by Baczek-Kwinta and Sala [68] in different sprouted seeds, where the authors did not find any correlation between antioxidant potential determined by the DPPH method and those observed in other antioxidant assays such as FRAP. Germination temperature, however, did not show a clear effect on sprouted lentil flour TAC measured by the different methods, with the exception of ABTS^•+^ radical scavenging activity (Figure 9d), which was clearly affected by the interaction between germination temperature and time. Increased ferric reducing potential was found at higher times (5 days) and lower temperature (15 °C) (Figure 9a). This trend was similar to that observed for TPS content (Figure 6), in close agreement with the findings of Zhang et al. [9], who also found a high correlation between FRAP and TPS results. ORAC assay (Figure 9b) showed similar behavior to that observed for FRAP, but the highest ORAC values were found at higher germination times (5 days) combined with medium temperatures (19–21 °C). DPPH^•^ radical scavenging activity, however, did not show any important changes as germination temperature and time increased (Figure 9c).

The similar trend obtained for TPS, insoluble and soluble phenolics (Figure 6, Figure 7 and Figure 8) and those obtained for the majority of antioxidant assays used suggest that phenolic compounds are the most important contributors to the antioxidant potential of sprouted lentil flour. The increased antioxidant activity in lentils after germination would respond to de novo synthesis of insoluble phenolic compounds due to the transport of soluble forms from vacuoles into the cell wall followed by transformation into insoluble phenolic compounds by formation of covalent bonds with cell wall components. Similarly, Strack et al. [69] reported that the increase in cell wall flavonoids in coniferae leaves occurred in parallel to the decrease of flavonoids and to the enhancement of deposition of kaempferol and quercetin derivatives in the cell wall.

Direct methods were also evaluated using DPPH^•^ and ABTS^•+^. The models showed a maximum at 21–22 °C and 3–4 days of germination, which were very similar results to those obtained by extractive methods. The values were significantly higher than those obtained using indirect (extractive) methods as was expected since the method evaluated soluble and insoluble phenolic compounds. The control showed values higher than the samples germinated following the same trend observed in extractive methods.

### 3.11. Starch Content and Glycemic Index of Sprouted Lentil Flours

**Figure 10:**
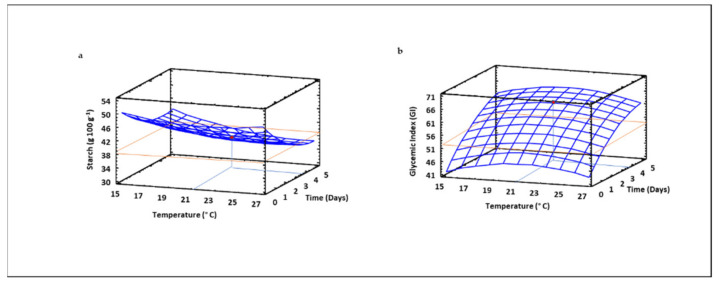
Three-dimensional response surface plots for the content of starch (**a**) and the glycemic index (**b**) as a function of the interaction between germination temperature and time. The red line corresponds with the control (ungerminated seed) and the red point with the value for optimum conditions (21 °C and 3.5 days).

The mathematical model obtained for starch content in sprouted lentil flour revealed that both germination temperature and time exert quadratic, linear and interaction effects on this parameter, and the model explained 52% of the changes in starch content in sprouted lentil flour (Table 2). The starch levels decreased as germination time increased (Figure 10a). As expected, starch is the major energy reserve used by the seed during germination, which is produced by mobilization of starch during the early stage of germination, resulting in the release of free sugars [41]. As germination time increased up to 72 h, the content of starch decreased probably due to the maximum activity of α-amylase, which slows down changes in starch content at longer germination times. These results agreed with those reported in lentil, pea, mung bean, oat and finger millet by other authors who found similar kinetics of starch degradation during germination [70,71,72].

The expected glycemic index (GI) was also evaluated in sprouted lentil flours and the RSM polynomial equation obtained showed that both germination temperature and time exert mainly linear effects on this parameter, although the quadratic and interaction terms of the equation were also significant (Table 2). Expected GI clearly increased as the germination time increased, due to the released reducing sugars as a consequence of starch degradation, while temperature had a less marked influence (Figure 10b). Similar enhancement of expected GI during germination was earlier found in lentil and barley [14,45,73,74].

### 3.12. Color of Sprouted Lentil Flours

**Figure 11:**
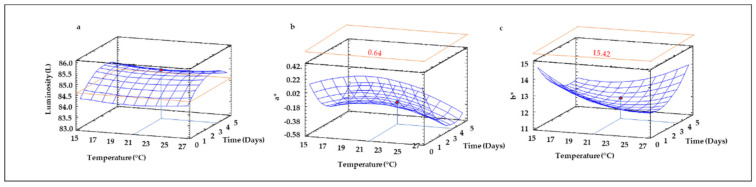
Three-dimensional response surface plots for CIE L*a*b* (CIELAB) parameter: Luminosity (**a**), a* (**b**) and b* (**c**) as a function of the interaction between germination temperature and time. The red line corresponds with the control (ungerminated seed) and the red point with the value for optimum conditions (21 °C and 3.5 days).

The color of sprouted lentil samples was measured by a colorimeter in the CIE Lab* scale and Hue and Chroma. As shown in Table 2, only linear terms of temperature and time were significant (*p* ≤ 0.05) in the polynomial equation describing the behavior of Luminosity value as a function of germination conditions, while for a*, b*, chroma and Hue, the quadratic and interaction terms also had a significant (*p* ≤ 0.05) influence. All the models were suitable for explaining color parameters as a function of germination time and temperature with the exception of that obtained for Luminosity, where the R^2^ value was low (0.15). Luminosity raised as germination time increased up to 3 days, and then suffered a slight decrease, while temperature increases did not notably modify the Luminosity of sprouted lentil flours (Figure 11a). In contrast, a* values decreased at higher germination temperature and longer times (Figure 11b), while b* values showed a more complex behavior, decreasing as germination time increased at lower temperatures while a higher temperature showed the opposite trend (Figure 11c). The results corresponding to a* and b* values agree with the MDA results (Figure 3) and the changes in both parameters are probably related to the increase of dark color in the lentil seeds during germination and the change of color from brown-red to green-yellow due to the growth of the germ.

### 3.13. RSM Optimization of Germination Conditions to Maximize the Quality of Sprouted Lentil Flours

**Figure 12:**
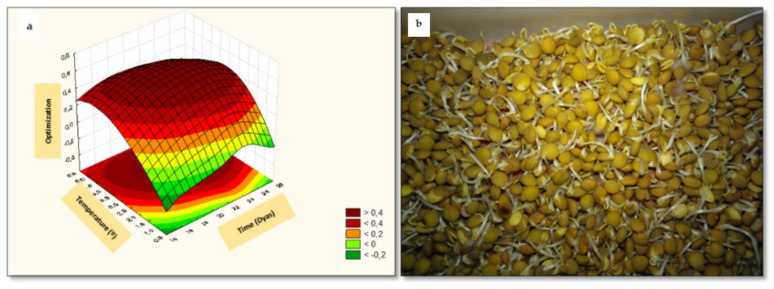
Three-dimensional response surface plots for desirability function (**a**) and image of germinated flour at optimal condition (**b**).

The desirability function (Figure 12) was used to find the optimal sprouting conditions (temperature and time) for the production of sprouted lentil flours with the highest nutritional and bioactive quality, and the lowest value of saturated fatty acids and expected GI. The desirability function integrates the individual responses of all the parameters evaluated and revealed that a germination temperature of 21 °C combined with a germination time of 3.5 days resulted in sprouted lentil flours with the highest nutritional and nutraceutical properties (overall desirability value at these conditions: 0.51).

The present study demonstrates that germination is a promising approach to obtain novel lentil flours with higher content of ascorbic acid, protein and fiber, as well as phenolic compounds and GABA.

The total phenol and antioxidants increased during the germination; however, the values were lower than those observed in ungerminated seeds. This reduction in phenols and antioxidants observed with respect to ungerminated seed has been studied by other authors, who reported that it may be the result of either leaching of these compounds into the soaking medium or poor extractability, as lower molecular weight phenolic compounds polymerize, thus becoming insoluble in water [75]. In addition, other authors have found than the activation of polyphenol oxidase enzyme during the soaking can promote the degradation and consequent loss of the polyphenol content and therefore reduce the antioxidant activity with respect to the control [76]. Other authors have also described the important effect of soaking and maintenance of humidity during the process [77] in seeds with similar external layer hardness and characteristic permeability such as soybean. In order to minimize the initial losses of polyphenols and antioxidant activity, the time reduction of soaking can be an effective method to avoid the observed solubilization and the control of humidity during the germination in air instead of capillarity in the chamber can also contribute to the observed losses.

## 4. Conclusions

Optimization using RSM allowed us to optimize germination conditions to increase the nutritional and health-promoting potential of sprouted lentil flours while minimizing the content of saturated fatty acids, phytic acid and glycemic index. RSM optimization of germination temperature and time using the desirability function revealed that the optimal process variables to maximize the nutritional, bioactive and quality properties of sprouted lentil flours were 21 °C and 3.5 days. However, it should be noticed that the study of other germination conditions beyond temperature and time might improve the reliability of RSM models, improving the potency of RSM optimization.

These results give novel insights into the development of nutritionally dense ingredients with improved health-promoting properties that can be applied as alternatives to commercial flours and also as novel ingredients for the development of innovative food products.

## Figures and Tables

**Table 1:** Central Composite Design (CCD) obtained by RSM, with two independent variables (time (t) in days and temperature (T) in °C), three center points and two-star points. The design was replicated.

Time (Days)	Temperature (°C)
2	17
3	21
3	21
2	25
4	25
3	21
1	21
5	21
4	17
3	15.3
3	26.7

**Table 2:** Predicted mathematical models and regression coefficients for the response variables studied in sprouted lentil flours as a function of germination temperature (T) and time (t). Response variables with R^2^ higher than 0.5 were included in the table.

Response Variables	Mathematical Models *	R^2^
Protein	12.83 + 1.12*T + 1.41*t − 0.02*T^2^ − 0.04*T*t − 0.09*t^2^	0.69
Carbohydrates	81.24 − 1.68*T + 0.52*t + 0.04*T^2^ − 0.04*T*t − 0.02*t^2^	0.72
Fiber	1.71 + 0.96*T + 1.49*t − 0.02*T^2^ − 0.04*T*t − 0.07*t^2^	0.63
MDA	−53.00 + 3.66*T + 9.47*t − 0.06*T^2^ − 0.12*T*t − 0.87*t^2^	0.96
Phytic Acid	1.26 − 0.02*T − 0.11*t + 0.0008*T^2^ − 0.009*T*t + 0.03*t^2^	0.81
GABA	650.94 − 68.61*T + 38.90*t + 1.78*T^2^ − 0.57*T*t − 2.47*t^2^	0.67
ORAC	−2370.23 + 494.28*T + 517.02*t − 11.22*T^2^ – 9.05*T*t	0.55
Starch	72.11 − 2.20*T − 2.48*t + 0.05*T^2^ – 0.08*T*t + 0.46*t^2^	0.52
GI	−4.40 + 4.72*T + 7.14*t − 0.11*T^2^ − 0.03*T*t − 0.55*t^2^	0.73
a*	−1.62 + 0.18*T − 0.24*t − 0.004*T^2^ − 0.006*T*t + 0.05*t^2^	0.62
b*	24.53 − 0.91*T − 1.93*t + 0.02*T^2^ + 0.06*T*t + 0.12*t^2^	0.82
Chroma	24.57 − 0.92*T − 1.93*t + 0.02*T^2^ + 0.06*T*t + 0.12*t^2^	0.82
Hue	1.46 + 0.18*T − 2.80*t − 0.004*T^2^ + 0.0003*T*t + 0.38*T^2^	0.77

* Models significance *p* ≥ 0.05 and lack of fit *p* ≤ 0.05 (ANOVA). Abbreviations: Malondialdehyde (MDA), γ-Aminobutyric Acid (GABA), Oxygen Radical Absorbance Capacity (ORAC), Glycemic index (GI).

## Supplementary Materials

The following are available online at https://www.mdpi.com/article/10.3390/foods10122924/s1. Table S1: Tentative identification of majority phenolic compounds in germinates by HPLC-ESI-QTOF-MS/MS under negative ion mode, Table S2: Quantification of the phenolic compounds was performed on a UPLC-MS/MS.
