# Supplementary material for: Development of Antioxidant and Nutritious Lentil (Lens culinaris) Flour Using Controlled Optimized Germination as a Bioprocess
Source: Foods. 2021 Nov 25;10(12):2924. doi: 10.3390/foods10122924 (PMC8700479; doi:10.3390/foods10122924)
Supplement: Supplementary file 1 [file foods-10-02924-s001.zip › foods-1469630-supplementary.pdf]

Table S1. Tentative identification of majority phenolic compounds in germinates by HPLC-ESI-QTOF-MS/MS under negative ion mode.

Compound	Observed $[M-H]^-$ (m/z)	Observed $[M-H]^-$ (m/z)	MS/MS (m/z)
**Quinic acid**	191.0545	191.0556	147.1, 173.0
**Gallic acid**	169.0158	169.0137	125.1
**2,3, Dihydroxybenzoic acid**	153.0207	153.0188	109.1
**4-hydroxybenzoic acid**	137.0257	137.0239	93.0
**catechin**	289.0724	289.0712	245.1, 203.1
**epicatechin**	289.0728	289.0712	245.1, 203.1
**p-coumaric acid**	163.0411	163.0395	119.1
**Ferulic acid**	193.0515	193.0501	149.1
**Luteolin-7-O-glucoside**	447.0931	447.0927	285.1, 151.0
**Q-3-glucoside**	463.0881	463.0877	301.1, 151.0
**Kaempferol-3-O-rutinoside**	593.1496	593.1507	285.1
**Luteolin 4'-O-glucoside**	447.0955	447.0927	285.1
**Luteolin**	285.0411	285.0399	151.1, 133.0
**Formononetin**	267.0516	267.0657	251.1

Table S2. Quantification of the phenolic compounds was performed on a UPLC-MS/MS. The results were expressed as μg g^{-1} of sample. *Analyses were carried out in triplicate extracts and target compounds were quantified using standard calibration curves of concentrations that ranged from 0.02 mg/mL–10 μg/mL

Temperature (°C)	Time (Days)	Type	Gallic acid (μg/mg)	2, 3 Dihydroxybenzoic acid (μg/mg)	Quinic acid (μg/mg)	4-hydroxybenzoic acid (μg/mg)	Ferulic acid (μg/mg)	Catechin (μg/mg)	Epicatechin (μg/mg)	Luteolin-7-O-glucoside (μg/mg)	Kaempferol rutinoside (μg/mg)	Luteolin-4'-O-glucoside (μg/mg)	Apigenin (μg/mg)	Q-3-glucoside (μg/mg)	p-Coumaric acid (μg/mg)
17	2	Insoluble	2.3±0.22	2.70±0.00	26.77±0.39	0.18±0.04	0.65±0.07	0.70±0.09	0.35±0.03	0.22±0.03	0.17±0.02	0.39±0.03	0.03±0.01	1,74±0.02	0.52±0.05
21	3	Insoluble	2.33±0.02	2.56±0.12	20.44±0.64	0.24±0.05	1.01±0.03	0.38±0.01	0.21±0.09	0.18±0.04	0.15±0.02	0.32±0.03	0.02±0.00	1.33±0.05	0.65±0.06
21	3	Insoluble	2.45±0.11	2.24±0.00	23.39±0.76	0.22±0.01	0.86±0.04	1.29±0.00	0,33±0.12	0.16±0.01	0.12±0.03	0.32±0.01	0.03±0.00	0.71±0.26	0.51±0.01
25	2	Insoluble	2.19±0.06	2.13±0.13	24.38±0.71	0.17±0.03	0.81±0.24	0.48±0.02	0.22±0.01	0.16±0.01	0.09±0.02	0.27±0.04	0.02±0.00	0.52±0.09	0.51±0.04
25	4	Insoluble	1.87±0.68	1.17±0.64	19.17±4.73	0.21±0.02	1.09±0.21	0.29±0.02	0.17±0.02	0.10±0.01	0.10±0.04	0.21±0.00	0.02±0.01	0.72±0.10	0.69±0.18
21	3	Insoluble	2.28±0.13	2.25±0.08	24.05±0.94	0.18±0.02	1.22±0.19	0.60±0.03	0.23±0.03	0.13±0.01	0.12±0.00	0.24±0.07	0.03±0.01	1.07±0.06	0.51±0.10
21	1	Insoluble	2.34±0.02	1.99±0.03	13.55±0.54	0.17±0.08	0.42±0.02	1.68±0.11	0.52±0.05	0.06±0.02	0.09±0.04	0.17±0.05	0.04±0.02	0.54±0.20	0.23±0.02
21	5	Insoluble	2.34 ±0,08	1.77±0.05	15.15±0.09	0.24±0.01	0.94±0.30	0.76±0.05	0.37±0.01	0.06±0.02	0.12±0.00	0.23±0.07	0.03±0.01	0.75±0.13	0.30±0.03
17	4	Insoluble	2.22±0,07	2.70±0.08	14.93±0.28	0.16±0.00	0.48±0.01	1.08±0.26	0.39±0.14	0.09±0.01	0.14±0.02	0.290.03	0.02±0.01	1,17±1.25	0.24±0.06
15.3	3	Insoluble	2.50±0,28	1.95±0.13	21.90±0.34	0.17±0.02	0.63±0.03	4.08±0.18	0.61±0.06	0.19±0.02	0.17±0.03	0,430.01	0.03±0.00	2.27±0.01	0.38±0.01
26.7	3	Insoluble	0.24±0,00	0.23±0.03	1.35±0.05	0.00±0.00	0.09±0.00	0.21±0.06	0.09±0.00	0.02±0.00	0.02±0.00	0.06±0.01	0.00±0.00	0.17±0.01	0.06±0.02
**CONTROL**		Insoluble	3.01±0,01	2.01±0.08	22.06±0.73	0.63±0.01	0.75±0.14	3.26±0.30	0.71±0.12	0.25±0.01	0.29±0.03	1.30±0.12	0.47±0.01	7.20±0.41	1.30±0.10
17	2	Soluble	0.00±0.00	0.00±0.00	5.89±4.05	0.00±0.00	0.00±0.00	0.05±0.04	0.00±0.00	0.00±0.00	0.02±0.02	0.05±0.03	0.00±0.00	0.00±0.00	0.00±0.00
21	3	Soluble	0.00±0.00	0.00±0.00	6.57±1.09	0.00±0.00	0.00±0.00	0.00±0.00	0.00±0.00	0.00±0.00	0.02±0.00	0.03±0.00	0.00±0.00	0.00±0.00	0.00±0.00
21	3	Soluble	0.00±0.00	0.00±0.00	6.28±0.77	0.00±0.00	0.00±0.00	0.00±0.00	0.00±0.00	0.00±0.00	0.01±0.00	0.02±0.00	0.00±0.00	0.00±0.00	0.00±0.00
25	2	Soluble	0.00±0.00	0.00±0.00	6.83±2.57	0.00±0.00	0.00±0.00	0.04±0.03	0.00±0.00	0.00±0.00	0.02±0.01	0.02±0.01	0.00±0.00	0.00±0.00	0.00±0.00
25	4	Soluble	0.00±0.00	0.00±0.00	5.38±1.74	0.00±0.00	0.00±0.00	0.00±0.00	0.00±0.00	0.00±0.00	0.04±0.04	0.02±0.01	0.00±0.00	0.00±0.00	0.00±0.00
21	3	Soluble	0.00±0.00	0.00±0.00	6.01±1.59	0.00±0.00	0.00±0.00	0.04±0.04	0.00±0.00	0.00±0.00	0.03±0.03	0.02±0.01	0.00±0.00	0.00±0.00	0.00±0.00
21	1	Soluble	0.00±0.00	0.00±0.00	12.86±1.99	0.00±0.00	0.00±0.00	0.05±0.04	0.00±0.00	0.00±0.00	0.04±0.00	0.03±0.01	0.00±0.00	0.00±0.00	0.00±0.00
21	5	Soluble	0.00±0.00	0.02±0.00	9.97±0.27	0.00±0.00	0.00±0.00	0.00±0.00	0.00±0.00	0.00±0.00	0.01±0.01	0.01±0.00	0.00±0.00	0.00±0.00	0.00±0.00
17	4	Soluble	0.00±0.00	0.02±0.01	5.80±0.88	0.00±0.00	0.00±0.00	0.00±0.00	0.00±0.00	0.00±0.00	0.01±0.01	0.02±0.01	0.00±0.00	0.00±0.00	0.00±0.00
15.3	3	Soluble	0.00±0.00	0.02±0.02	9.32±0.47	0.00±0.00	0.00±0.00	0.05±0.04	0.00±0.00	0.00±0.00	0.04±0.01	0.03±0.01	0.00±0.00	0.00±0.00	0.03±0.00
26.7	3	Soluble	0.18±0.00	0.06±0.01	5.06±0.38	0.00±0.00	0.00±0.00	0,15±0.00	0.05±0.00	0.00±0.00	0.02±0.01	0.03±0.01	0.00±0.00	0.00±0.00	0.03±0.00
**CONTROL**		Soluble	0.03±0.01	0.02±0.00	13.60±1.75	0.00±0.00	0.00±0.00	0.08±0.01	0.04±0.00	0.00±0.00	0.01±0.00	0.04±0.01	0.00±0.00	0.00±0.00	0.04±0.00
